# COVID-19 and Health-Related Authority Allocation Puzzles

**DOI:** 10.1017/S0963180120000468

**Published:** 2020-06-08

**Authors:** MICHAEL DA SILVA

**Keywords:** COVID-19, healthcare policy, health law and policy, justifiable authority allocation, emergency powers

## Abstract

COVID-19-related controversies concerning the allocation of scarce resources, travel restrictions, and physical distancing norms each raise a foundational question: How should authority, and thus responsibility, over healthcare and public health law and policy be allocated? Each controversy raises principles that support claims by traditional wielders of authority in “federal” countries, like federal and state governments, and less traditional entities, like cities and sub-state nations. No existing principle divides “healthcare and public law and policy” into units that can be allocated in intuitively compelling ways. This leads to puzzles concerning (a) the principles for justifiably allocating “powers” in these domains and (b) whether and how they change during “emergencies.” This work motivates the puzzles, explains why resolving them should be part of long-term responses to COVID-19, and outlines some initial COVID-19-related findings that shed light on justifiable authority allocation, emergencies, emergency powers, and the relationships between them.

Several recent COVID-19-related controversies independently raise unique concerns but jointly highlight the need to reflect on a more basic question: How can and/or should authority, and thus responsibility, over healthcare and public health law and policy be allocated within (and possibly across) countries? The pandemic is, of course, a complex challenge that requires responses from various actors and may require new approaches to collaboration between different governments and nongovernmental actors.^1^ Yet the question of who should have primary law- and policymaking authority over particular subjects (in the sense of being able to make final decisions free from interference from other actors) is analytically prior to determining how such “collaboration” should take place. It is also central to ongoing disputes in the real-world circumstances in which ideal collaboration cannot be assumed. Although the controversies take different forms across the globe, the underlying issues are perfectly general, arising in “federal” countries, like the United States and Canada, and more “centralized” ones, like France and Israel, where all formal powers rest with a single level of government but administrative authority rests with more “local” actors who often have broad discretion to act without *close* oversight. As I hope to show below, analysis of the basic issues is also independently important for our understanding of the nature of justifiable authority allocation and of emergencies.

One cannot resolve all COVID-19 controversies in a single work but explaining how many pose puzzles for our understanding of authority allocation *and* emergencies should help us better understand the conceptual underpinning and stakes of the debates and help resolve them. This work thus identifies the puzzles. I call them “authority allocation” puzzles because they focus on basic issues of how to justifiably, let alone ideally, allocate “powers” over particular subjects. I do not mean to directly contribute to debates on whether the wielders of such powers are “legitimate authorities” in the sense of providing people with content-independent reasons for action.^2^ I instead wish to contribute to debates on how to allocate law- and policymaking “powers” and whether emergencies provide reasons to consider new principles of allocation or apply existing principles in new ways.^3^ I focus on authority over healthcare and public health law and policy because decisions made in the name of these subjects are most central to COVID-19 debates and the principles for allocating authority over these domains in particular are long overdue for reconsideration in any case.^4^ COVID-19-based cases that raise and motivate the puzzles provide initial indications on how best to resolve them that I will mention briefly, but they are merely suggestive. Further works must more conclusively resolve the puzzles.

Both authority allocation puzzles relate to the difficulties of determining who should make decisions in health-related law and policy domains. To begin, consider recent controversies about who should make decisions about the allocation of scarce resources, including personal protective equipment (PPE) and ventilators. This issue has arisen in nearly every country impacted by COVID-19, but I will begin with North American examples, which are particularly useful for two reasons. First, authority in the United States and Canada is always shared between “federal” and “state” governments with primary authority over healthcare and public health resting at the state level.^5^ Although authority allocation questions arguably arise for all governments—after all, Paris cannot make every decision about healthcare or public health even in centralized France where all formal authority rests with one government—North America has a long history of attempts at “principled” allocation that one can use to identify and analyze reasons for placing authority over different subjects at different levels. North American countries’ largely exceptional decision among “federal” nations to allocate primary authority over healthcare and public health to the states makes it a good case study in one approach to allocation.^6^ Second, COVID-19 arrived in North America later than many countries, which provided actors at various levels of government in the United States and Canada with time to use their existing powers to attempt to contain the pandemic.^7^ Some data on how best to respond and even which levels of government could best respond already existed when COVID-19 arrived in North America. This combined with existing polarization to make authority claims key to debates in those countries.^8^ Those debates contain claims that require and help explain the practical stakes of this inquiry.

The authority and responsibility for securing and distributing these essential resources are subjects of fierce debate at multiple levels of government. The American federal government has, for instance, been criticized for attempting to maintain control over the allocation of federally-sourced PPE.^9^ Although one can question that decision—and discussion of a federal “stockpile” in opposition to a state one can be read as suggesting an antagonism at odds with national solidarity that warrants questioning^10^—the idea that the federal government is best placed to allocate these resources remains compelling. This is true wherever unjust distributions obtain. Some view North American federal governments’ particular failures to secure adequate resources prior to the pandemic reaching the continent as favoring North American countries’ “decentralized” approach to healthcare and public health law and policy, but distribution of the goods those countries do possess is unequal across their constituent units (states/provinces) and inefficient in the sense of ensuring PPE are where they are most needed.^11^ Although that problem too may be partially attributable to existing federal institutions, it is not wholly attributable to them. Competition between states with unequal (and weak relative to the federal equivalent) bargaining power surely also contribute to the disparities. A strong response by a well-constituted federal government that leverages its own notable resources could efficiently resolve unjust disparities. This issue is then mirrored at more local levels. Whether the allocations of PPE and ventilators within hospitals should be a matter of federal, state, or municipal policy is a live question. Even those who think allocation should be left to clinical judgment must decide which level of government should have the authority to leave the decisions to physicians.^12^

At the same time, COVID-19 occasioned claims for greater decision-making authority by states, municipalities, and even sub-state nations (e.g., Indigenous groups and other groups who share characteristics of “nations” but lack their own countries) due to perceived lacks in federal or state coordination and/or consideration of more local needs.^13^ Whatever the merits of particular responses, the empirics of the COVID-19 pandemic support claims that at least some groups should have more authority over healthcare and/or public health law and policy in particular. The differential impact of COVID-19 across states and geographical and technical scope of emergency management may support strong federal “powers” in the relevant domains, but the COVID-19 pandemic also identifies local concerns that may be best-addressed at lower levels and underlines municipal and sub-state actors’ abilities to innovate.^14^ Consider ongoing debates about the regulation of physical distancing measures. Battles between federal and state governments over who can decide when to impose and lift social distancing requirements are, perhaps, uninteresting for constitutional scholars.^15^ Yet questions about which government should be able to impose restrictions in the name of public health should interest all stakeholders.

Recent controversies about border control also highlight these questions. For instance, the closure of the United States–Canada border was a symbol of the pandemic’s seriousness.^16^ Canadian provinces have since attempted to restrict travel between provinces.^17^ Necessity-based arguments for these claims are intriguing regardless of their constitutional pedigree.^18^ Some municipalities were then placed under de facto quarantine with travel to those areas restricted by provincial laws.^19^ This phenomenon is not specifically Canadian—and not always a topic of provincial control. A challenge to a decision by the Israeli government to restrict travel to a city there has already been heard by the Supreme Court of Israel.^20^ Still other cities have called for greater autonomy over decisions to close their borders. For instance, remote coastal communities in British Columbia (BC)—many of which double as Indigenous communities that should possess at least some self-government rights under Canadian constitutional law—sought to restrict access to their lands absent provincial declarations that are legally required under BC law.^21^ Authority allocation analysis is necessary to test the legitimacy of these varying claims to “powers.”

Less dramatically, but equally (if not more) importantly (for at least most people), even a cursory glance at the newspapers suggests that COVID-19 raises questions about the extent to which localities should be able to tailor restrictions to reflect different needs and/or priorities.^22^ Municipal decisions to close public parks and other municipal venues in North America were largely viewed as both legally grounded and necessary to combat the spread of COVID-19. Other authority claims are, however, potentially more challenging. For instance, public education is clearly within provincial jurisdiction in Canada. Yet English school boards in Canada’s most COVID-19-impacted city, Montreal, refused to open schools on the timeline set by the “nationalist” French-Canadian Government of Quebec, whose base largely lies outside Montreal.^23^ COVID-19 demands scrutiny of whether cities should be able to make decisions about these mandatory services. Claims that the provinces lack knowledge of and/or solidarity with the people of Montreal, undermining their authority to regulate, reflect philosophical claims that municipalities should have greater authority over different subjects and demand scrutiny.^24^

Still other recent cases raise broader questions about whether cities should be able to set their physical distancing policies contrary to central and/or state authorities’ aims. For instance, most cities have clear powers over zoning and licensing of public spaces that they have used to, for instance, create pedestrian walkways and ban large gatherings. Yet the scope of these powers when they run contrary to federal or state desires is contestable. Consider several cities’ continued closing of concert venues even after Missouri “reopened” to public performances.^25^ Missouri has not ordered the reopening of the venues, so no formal conflict exists at present. But continued closures run contrary to the economic rationale underlying the state decision. Whether cities should be able to make their own decisions on when to open venues remains questionable.

Each of these controversies includes competing claims to authority over at least aspects of healthcare and public health law and policy that jointly present a pair of puzzles requiring reconsideration of how to justifiably allocate authority in those domains. My intuitions about which claims can be justifiably or should be accepted are not uniform. I am, for instance, moved by distributive justice concerns suggesting that federal authorities are best-placed to distribute scarce resources and epistemic concerns suggesting that cities may be best-placed to make decisions about how to allocate them—and even democratic accountability concerns related to the break between the number of city-dwellers impacted by the pandemic and the number of representatives that they have in federal or state governments managing responses.^26^

Conflicts in intuitions about where to place authority are not easily resolved by simply appealing to an existing principle for allocating authority and may even justify, if not require, allocating authority to nontraditional candidates for authority, like cities or sub-state nations. Giving lexical priority to a centralizing principle like “coordination” or a decentralizing one like “local concern” fails to account for competing intuitions about cases and raises questions about how to resolve conflicting claims on the basis of those values. For instance, appeals to the need for “coordination” arguably fail to take advantage of the considerable epistemic resources of local communities that could be leveraged to produce better results overall, potentially explaining why some local communities have been hit harder by COVID-19 than others. Moreover, “coordination” alone no longer favors countries’ domestic central governments alone. Even if one is willing to accept the costs of “coordination,” *global* inequities and the global nature of the relevant coordination problem may equally favor centralization towards a global authority. The United Nations’ response to COVID-19 has been questionable, but COVID-related recent advocacy for a “world government” is unsurprising.^27^ Puzzles about how to account for prima facie compelling claims by these new entities and which principle(s) best resolve competing claims will remain even if we find out that “coordination” matters most during pandemics.

An appeal to local strengths purporting to justify providing cities with unique domains of authority leaves parallel issues without satisfactory resolutions. It could, for instance, constitute an ad hoc resolution of the issue that gives up on basic concerns with distributive justice by making access to some goods conditional on local preferences many local residents may not share. It certainly fails to account for the way in which other groups, like sub-state nations, also face unique needs that equally warrant providing them with powers and, further, possess unique preferences that members view as more central to their identity than their place of residence.^28^

Simply dividing “healthcare and public health law and policy” into various distinct law and policy domains could help address this issue, but it is not clear that one can do so in a principled manner that captures intuitions about the cases above. The underlying tensions arise in both broadly defined areas like “healthcare and public health law” and narrowly defined domains like “ventilator distribution.” In each case, general principles for justifying allocations support competing authority claims. For instance, compelling principles seem to promote multiple candidates for authority over ventilator distribution. “Epistemic” concerns equally favor federal knowledge of the “big picture” and state and local actors’ local knowledge. Outcomes for allocations favoring either distribution are not uniform. Attempts to then carve up “public health policy” so that “travel restrictions” fall under primary federal control but “school openings” fall under local control require a principle of selection I have yet to identify. Even those who accept that it *could* be identified should agree that scrutiny of existing allocation principles is needed. The clear lack of fit between principles, institutions for realizing them, and intuitions demands it.

These concerns jointly present a pair of related puzzles. The first examines how one can justifiably allocate authority over healthcare or public health law and policy. This puzzle asks, “Which principles are relevant to the allocation of authority over particular claims and what is the range of justifiable options for weighing competing principles?” It seeks to identify which entities can possess the relevant authority according to our best principles and what to do in cases where multiple justifying claims exist. The second puzzle examines whether pandemics’ exceptional nature can justify and/or requires reallocation of some healthcare and public health law and policy-related “powers” during similar emergencies. It asks, “Are emergencies ‘exceptional’ in a way that justifies or requires different principles for allocation?” Federal governments in countries with multiple levels of governance often possess “emergency powers” they can use to act in the name of emergency management in domains where they normally lack authority.^29^ Whether these principles are justified by the “exceptional” nature of emergencies has long been a source of controversy.^30^ COVID-19 highlights an often-overlooked complication for traditional approaches to the issue. As discussed above, principles justifying federal “emergency” powers may require allocating authority to other entities. Whether desired allocations of authority to those entities are justified, or even required, due to emergency exigency or some other principle, is not only important for assessing existing constitutional divisions of powers in federal countries. It also speaks to the scope and nature of emergencies and emergency powers.

COVID-19 stresses the need to resolve the first puzzle and the importance of considering a wider variety of entities as potentially justified wielders of the relevant authority to do so. Every country must decide how to allocate authority. Again, even countries that formally contain a single level of government, like France and Israel, must devolve some administrative decisionmaking to other levels. Principles promote different allocations. For instance, interests in coordination can support federal control and interests in flexibility can support more local control. Authority allocation decisions purportedly based on these principles have profound impacts on access to healthcare and health outcomes,^31^ failing to fully realize those principles. Existing (if ever-changing) COVID-19 data underscores differences between areas of decentralized countries, if not differences between centralized and decentralized countries.^32^ At the same time, better outcomes in some cities suggest that principles supporting state control could be better instantiated through municipal control.^33^ Calls for greater municipal authority over some subjects have thus appeared even in paradigmatically centralized France.^34^ There are good (prima facie) reasons to consider their potential validity, but further analysis is needed.

Compelling claims to authority and actions by cities and sub-state nations then provide prima facie cases for their status as justifiable wielders of authority. This vindicates past claims that cities and sub-state nations should be included in theoretical examinations of how to allocate authority within countries.^35^ For instance, the same epistemic concerns that purport to justify state control now appear to equally justify municipal or sub-state control, at least prima facie. Seattle and San Francisco are notable examples of cities leveraging local knowledge to great effect while the impact of COVID-19 on Montreal raises questions about whether Quebec’s provincial government is best-placed to make decisions for that city (and Montreal’s community groups help ensure access to scarce resources there absent provincial action).^36^ Indigenous nations, like those in coastal BC, make good cases for sub-state national control.^37^ Analyses of authority allocations thus should not be limited to federal and state governments alone absent some explanation of why the relevant principles do not apply to the other groups. But even that finding suggests that a complete account of justified authority must address cities and sub-state nations as potential authorities. COVID-19 still occasions a puzzle about how to justifiably allocate authority and stresses the need to consider new entities to resolve that puzzle.

COVID-19 also raises the second puzzle about whether general authority allocation principles apply during emergencies or give way to emergency “exceptionalism.” Some political entities allowed others to go beyond their traditional boundaries of authority during the pandemic. For instance, the aforementioned Government of Quebec invited the federal army into the province to help run long-term care homes.^38^ The extent to which pandemics justify these and other deviations from traditional allocations of authority speak to the nature of justifiable authority allocation. They also speak to the nature of emergencies and their impact on authority allocation questions. For instance, municipal and state border restrictions are likely illegitimate violations of freedom of movement outside pandemic conditions.^39^ Even if they could be justified qua rights infringements during pandemics, questions about whether provinces or municipalities should be able to make those restrictions during pandemics remain.

Even if one could resolve *those* issues, further questions about whether pandemics warrant reallocating authority and require different applications of the relevant principles would remain. Are, for instance, local knowledge and needs more important during a pandemic that disproportionately affects members of localities with a shared set of features? Local knowledge, needs, and disproportionate impact are often cited as reasons that cities should have power over particular policy areas.^40^ Cities have been hit much harder by COVID-19 and arguably have better knowledge of local needs and resources capable of addressing those needs as well as a better understanding of how density complicates general physical distancing rules. If these factors cannot justify providing them with general powers over healthcare and public health law and policy, they might still justify exceptional powers during emergency that is primarily located in cities, like COVID-19. At the same time, local control in these circumstances might exacerbate distributive justice problems, requiring a federal response. Cities’ use of existing powers has not led to uniformly strong responses in North America.^41^ Whether expanded powers would produce any better results is unclear but concerns about inequities are not easily diffused.

Full resolution of either of these puzzles requires several works, but COVID-19 raises them in interesting ways and even a cursory overview provides initial insights into their resolution. Notably, for instance, each principle at issue in the cases above appears in wider literatures on authority allocations in general and “healthcare federalism” in particular. The conflicting values raising apparent dilemmas in those cases likewise mirror tensions in wider literatures.^42^ This suggests that pandemics do not pose unique concerns in this area but merely raise existing issues. If this is so, the “exceptional” nature of pandemics does not raise exceptional principles of allocation and/or problems with same. Although federal governments are often provided with exceptional powers during emergencies, that allocation arguably follows a general principle suggesting that federal governments can justifiably legislate over matters that cannot be addressed at lower levels, which paradigmatically include national emergencies.^43^ Where, in turn, COVID-19 stresses the need to consider other entities as potential wielders of authority, new prima facie cases for authority do not rely on emergencies’ “exceptional” nature.

Pandemics instead appear to provide extreme examples of the strengths and weaknesses of different allocation options and the cost of different tradeoffs between options. The way in which federal coordination concerns appear particularly important in pandemics suggest that allocating at least some emergency powers to federal governments, as is common in federal countries, has some merit.^44^ But the motivating coordination principle is not unique to emergencies and competing intuitions about cases above suggest that that principle may not be conclusive of how authority is best allocated during pandemics. The justification accordingly does not appear to stem from the exceptional nature of “emergencies” alone. At the same time, pandemic conditions do not appear to provide a conclusive all-things-considered case for providing cities with greater authority over healthcare and public health authority. Again, COVID-19 clearly provides evidence of local needs and resources that constitutes a prima facie case for greater municipal control, which itself requires considering cities in any complete account of justified authority, but those principles do not uniquely select cities *even in pandemic conditions.* COVID-19 also highlights some issues with municipal control. It has, for instance, emphasized the importance of solidarity for combatting public health issues. Given demographic sorting, providing more power to cities may be viewed as providing more power to “liberals” and undermine solidaristic practices outside cities.^45^ Where solidarity is also central to successful public healthcare, this concern may also apply to municipal healthcare authority.^46^ Solidarity requires “the recognition of similarity, and its prioritisation (at least in one respect) over difference.”^47^ Reifying difference along an axis with politicized features may undermine it.

Conclusive judgments on whether worries about increased federal or municipal powers are well-grounded or related tradeoffs are acceptable are beyond the scope of this inquiry. The point here is that the concerns and tradeoffs in the federal and municipal cases are largely just enhanced versions of those in “regular times.” Pandemics alone thus appear unable to justify reallocating powers. One cannot simply point to the existence of an “emergency” to trigger new criteria for evaluating authority claims but must examine how existing principles apply in a given setting. Scrutiny of general principles and their interaction in pandemic conditions is required. Given the porous, largely socially constructed nature of “emergencies,” this is likely a happy result: appealing to “emergency” to justify exceptional powers too easily risks overly strong federal governance.^48^ But these early findings are mere indications of potential solutions.

More work is needed. Indeed, analysis of different authority allocation options is important independent of the pandemic. Although many have long imagined that the reasons for particular authority allocations within countries are justified, applying basic principles to healthcare and public health will not immutably select particular allocations. The general principles are often scrutinized,^49^ but much less has been written about their application to the subjects at hand. Health-related stakeholders often take existing allocations for granted. For instance, although impressive empirical research examines how choices to allocate powers at federal or state levels impact access to healthcare and health outcomes, most pre-pandemic works on healthcare federalism took existing authority allocations as parametric and detailed their consequences.^50^ Even theorists working on health justice often do not examine the impact of allocation choices *within* countries.^51^ I have worked on these issues for some time and, a few borderline cases aside, I am only aware of two major *philosophical* analyses of healthcare “federalism.”^52^ Examinations of when it is justifiable to allocate authority to entities other than federal or state governments are largely nonexistent. Work on cities and sub-state nations rarely, if ever, focuses on healthcare or public health authority.^53^ Reconsideration is already long overdue. Ethicists are well-suited to contribute to it by examining and weighing principles.

COVID-19 offers a unique opportunity to reconsider these basic questions and stresses the need to do so, even if emergencies ultimately do not introduce unique principles or problems of allocation. The shock of COVID-19 and return of authority questions to the political sphere may offer opportunities to reallocate powers, but this analysis is important regardless of whether political conditions offer strong opportunities to reallocate powers in the long-term.^54^ For instance, recent political rhetoric about the boundaries of authority necessitates and provides an opportunity for reconsideration of these issues while actions taken pursuant to real and imagined authority help further underscore the actual impacts of allocation decisions. These concerns jointly necessitate reconsideration of basic allocation questions, for the sake of understanding the legitimacy of real actions and justification and real-world impact of authority allocation decisions. Further analysis of the relevant principles and their interaction in and outside pandemic conditions should provide insight into overlooked questions concerning justified authority, the potential wielders thereof, and the nature and scope of emergency “exceptions.” Ethicists should not leave this important work to political scientists and lawyers alone.^55^

## Notes

1. On the collaboration issue, see for example, Paquet M, Schertzer R. COVID-19 as a complex intergovernmental problem. *Canadian Journal of Political Science* 2020;Online First:1–5.

2. See, for example, any basic work on “authority” or “legitimacy,” including the collection of classics in Raz J , ed. Authority. New York: New York University Press; 1990. Although much has changed in the literature since 1990, the basic puzzle remains. More recent Stanford Encyclopedia of Philosophy entries on “Authority,” “Political Authority,” and “Legal Obligation and Authority” are all good on this. More detailed citations seem inapt where this is not my focus.

3. For overviews of historical and contemporary positions on authority allocation, see Karmis D, Norman W, eds. *Theories of Federalism: A Reader.* New York: Palgrave; 2005 or Føllesdal A. “Federalism.” *Stanford Encyclopedia of Philosophy*; 2003/2018; available at https://plato.stanford.edu/entries/federalism/. For an excellent collection of recent essays, see Fleming JE, Levy JT, eds. *NOMOS LV: Federalism and Subsidiarity.* New York: New York University Press; 2014. Note, however, that few works in any of these fields focus on how to allocate health-related powers in particular. I cite some of the only works on that topic below. Works on “emergency” powers are, of course, numerous. Works on how public health emergencies impact the distribution of authority are less common. Even works in this less common genre (e.g., Attaran A, Wilson K. A legal and epidemiological justification for federal authority in public health emergencies. *McGill LJ* 2007;52:381–414; available at http://jcb.utoronto.ca/publications/documents/AttaranWilson_2007_McGillLawJ_52_2_381.pdf.)) tend to focus on federal powers alone. My puzzles suggest these helpful works only address part of the issue.

4. See, for example, the final two paragraphs of this article.

5. Although states maintain primary authority over health-related powers in the United States, federal taxation and spending powers—and the “General Welfare” power—have, of course, allowed for some shared federal governance in this area. On Canada, see for example, Flood CM , Lahey W , Thomas B. Federalism and health care in Canada: A trouble romance? In: Peter Oliver , ed. The Oxford Handbook of the Canadian Constitution. Oxford: Oxford University Press; 2017 at 449 . For a dated text that discusses many basic cases, few of which have been superseded by non-*Patient Protection and Affordable Care Act*-based concerns, see Banting KG, Corbett SM, eds. Health Federalism and Policy. Montreal, Canada: McGill-Queen’s University Press; 2002 .

6. On that exceptional status, see for example, Popelier P , Cantillon B. Bipolar federalism and the social welfare state. Publius 2013;43(4):626–47. Note, however, that the location of “primary” authority may exist on a continuum and that trends toward “decentralization” may complicate the sense in which they remain genuine outliers.

7. Dating on this may remain contestable, but both at least faced *high* numbers only after Asian and European spread.

8. See the examples of key controversies discussed throughout this paper.

9. For a media report on one of the more controversial claims to federal authority, see Mazza E. Jared Kushner ripped for saying ‘Our Stockpile’ isn’t meant for states to use. *The Huffington Post* 2020 Apr 3.

10. As PolitiFact notes, https://www.politifact.com/article/2020/apr/03/fact-checking-jared-kushners-comments-national-sto/, Kushner’s controversial phrasing needed to be put “in context” and can be read as a simple statement that the federal government should decide how to distribute scarce resources. Although this charitable reading is more compelling, the phrasing remains unfortunate and at least opened the door to some of the criticism in note 9. My comments in the rest of this paragraph are not meant to be read as a justification for the actions of particular existing federal governments but only as a case for allocating authority to a well-constituted one.

11. This point has been widely documented and discussed. For example, Soergel A. States competing in global Jungle for PPE. *U.S. News& World Report* 2020 Apr 7; available at https://www.usnews.com/news/best-states/articles/2020-04-07/states-compete-in-global-jungle-for-personal-protective-equipment-amid-coronavirus (last accessed 14 May 2020).

12. For a brief, widely-shared essay from early in the pandemic that mentions the patchwork of regulations on ventilator distributions and goes on to criticize guidelines that did exist at the time, see White DB, Lo B. A framework for rationing ventilators and critical care beds during the COVID-19 pandemic. *JAMA Network* 2020;323(18)1773–1774; available at https://jamanetwork.com/journals/jama/fullarticle/2763953.

13. Although the American experience largely tracks traditional divisions of authority between the federal and state levels (with some discussion of increased municipal control), note potentially less politically polarized yet still theoretically difficult cases in other countries, from local boards seeking to set their own terms for “return to school” in Canada’s most COVID-impacted city (Meagher J. Quebec English school boards say they will decide when to reopen. *Montreal Gazette* 2020 May 1.) to Indigenous nations and other coastal communities seeking to set the terms of access to parts of British Columbia absent provincial authority to do so (Marlan T. Death strikes coastal first nations, communities talk of ‘thwarted’ attempts to protect themselves. *Capital Daily* 2020 Apr 25.). Further examples below (e.g., from France) show that this extends outside North America. Indeed, one merely needs to glance at the headlines each day to see new examples of contested authority in many countries.

14. For two examples of praise for local responses, see Berman R. The city that has flattened the coronavirus curve. *The Atlantic* 2020 Apr 12 and Duhigg C. Seattle’s leaders let the scientists take the lead. New York’s did not. *The New Yorker* 2020 Apr 26; https://www.newyorker.com/magazine/2020/05/04/seattles-leaders-let-scientists-take-the-lead-new-yorks-did-not.

15. The relevant authorities are generally clear as a matter of law in both countries, as the quick response to President Trump’s claims to “total authority,” exemplified in, for example, John A. Does Trump have ‘total authority‘ during the coronavirus outbreak, or any other time? *Los Angeles Times* 2020 Apr 18, helped make clear.

16. See, for example, BBC News. US–Canada border to close amid virus crisis. *BBC News* 2020 Mar 18.

17. For a good overview, see MacGregor S. Canada’s Provinces introduce new coronavirus travel regulations to limit domestic travel. *Forbes* 2020 Apr 3.

18. Technically, *Canadian Charter of Rights and Freedoms*, Part I of the *Constitution Act, 1982*, being Schedule B to the *Canada Act 1982* (UK), 1982, c 11, s 6 only provides inter-provincial travel rights to “move to and take up residence in any province” and “pursue the gaining of a livelihood in any province” and even these rights are subject to a limitation clause in s 1 of the *Charter.* But constitutional rights concerns at least make sense in this context. Whether provinces have the *jurisdiction* to close borders remains a good question independent of rights concerns.

19. Note, for example, police checkpoints discussed in Boshra B. Quebec to gradually remove travel restrictions between most regions starting May 4. *CTV News* 2020 Apr 29. Cities in that province were viewed as having their own authority to restrict travel to and within their borders, though Montreal only suggested such restrictions and did not enforce them; Elliot T. Mayor Plante is now asking everyone to stay in their own neighbourhoods. *MTL Blog* 2020 Apr 3; available at https://www.mtlblog.com/news/canada/qc/montreal/montrealers-should-stay-in-their-own-neighbourhoods-mayor-plante-says (last accessed 14 May 2020).

20.
*Yedidya Loewenthal, Adv v Prime Minister,* HCJ 2435/20 (Isr).

21. See, for example, note 13. My description of self-government rights here is descriptive, not normative.

22. For these rationales as justifications for general municipal control over at least some subjects, see, for example, Weinstock D. Cities and federalism, at 259 In: See note 3, Fleming, Levy 2014; King L. Cities, subsidiarity, and federalism, at 291. In: Fleming, Levy 2014, at 259. For evidence of unique local needs and values and of local knowledge of the issues and resources needed to resolve them in the pandemic context, see for example, Grillo M. Activists in Montreal area hardest hit by COVID-19 distribute face coverings. *CTV News* 2020 May 2.

23. See note 13, Meagher 2020. Whether they will open per since-delayed provincial orders is a live question today.

24. See note 22.

25. Minsker E. Missouri Governor says concerts can resume, cities keep venues closed. *Pitchfork* 2020 May 3; available at https://pitchfork.com/news/missouri-governor-says-concerts-can-resume-cities-keep-venues-closed/ (last accessed 14 May 2020).

26. This admittedly dense passage builds on the applications of principles in sources; for example, see notes 3 and 22.

27. For a criticism of the United Nations’ response, see Brunnée J. The UN’s relative silence speaks volumes about the U.S.’s failure to lead. *The Global and Mail* 2020 Apr 13. For the world government advocacy, see for example, the widely-shared blog post online at https://theweek.com/articles/905864/coronavirus-case-oneworld-government. This case builds on a longer tradition, summarized in Lu C. World Government. *The Stanford Encyclopedia of Philosophy* 2012; available at https://plato.stanford.edu/entries/world-government/.

28. I have a piece under review that evaluates potential sub-state national control. Google Data (discussed here https://globalnews.ca/video/6781249/coronavirus-outbreak-google-data-shows-quebec-has-best-social-distancing-compliance-in-north-america) highlighting Quebec’s remarkable compliance with social distancing norms also suggests that sub-state nations may engender the kind of “loyalty” needed to secure widespread compliance with physical distancing norms. Cities traditionally do not engender such loyalty (though King at 316 and de-Shalit A. Cities and Immigration: Political and Moral Dilemmas in the New Era of Migration. Oxford: Oxford University Press; 2019 champion some forms of “urban loyalty”). Greater loyalty to that identity further suggests that people view that group as the one “most affected” by different policies. The Indigenous national claims in the coastal case above may, in turn, be even stronger than other municipal claims. See for example, Boyer Y. Moving Aboriginal Health Forward: Discarding Canada’s Legal Barriers. Vancouver: University of British Columbia Press; 2015 for unique Indigenous issues.

29. See my piece in the last note for a detailed discussion. Note, for instance, the constitutionally-acceptable *National Emergencies Act*, 50 USC c 34 sec 1601 et seq or Canada’s equivalent *Emergency Management Act*, RSC, 2007, c 15.

30. Legal scholars often discuss this; Dyzenhaus D. The Constitution of Law: Legality in a Time of Emergency. Cambridge: Cambridge University Press; 2006 ; Lazar NC. States of Emergency in Liberal Democracies. Cambridge: Cambridge University Press; 2009 ; Ramraj VV , ed. Emergencies and the Limits of Legality. Cambridge: Cambridge University Press; 2009 ; Loevy K. Emergencies in Public Law: The Legal Politics of Containment. Cambridge: Cambridge University Press; 2016 ; Stacey J. The Constitution of the Environmental Emergency. Oxford, UK: Hart, 2018; etc.

31. See note 5, Banting, Corbett 2002 is a classic on this. For a recent case study on impact of federalism on health in the United States, see Michener J. *Fragmented Democracy.* Cambridge: Cambridge University Press; 2018. Both texts are representative of a wider field. But the impact of federalism in particular on social policy, including the development of welfare systems in “decentralized” countries, itself takes numerous forms and depends on various factors; Weaver RK. Policy dynamics in federal systems: A framework for analysis. Publius 2019;50(2):157–87.

32. This data is highly contested, but even a cursory glance at the headlines clarifies this much. For a nonacademic but useful high-level discussion of this issue, see Murtha J. COVID-19: Who’s in charge here? *MDLINX* 2020 Apr 7. Note, however, that nothing in my arguments depends on the truth of claims in this nonacademic source. Some data suggesting different outcomes is notable, but the analysis remains important given the pre-pandemic impacts noted in the last note and political rhetoric suggesting that differences justify reallocating authority suffices to justify my analysis even if the underlying data ultimately does vindicate the relevant claims.

33. Recall, for example, the celebrated cases of Seattle and San Francisco highlighted in note 14.

34. Onishi N, Méheut C. Pandemic shakes France’s faith in a cornerstone: Strong central government. *The New York Times* 2020 Apr 29.

35. Recall for example, the sources in note 22. Both texts in note 22 and the overviews in works in note 3 also mention several other works on the relevant issues; for examples, see note 22, Weinstock at 260 and note 22, King at 295n10. Bauböck R. Reinventing urban citizenship. Citizenship Studies 2003;7:139 is one classic source cited therein. For a more recent text, see note 28, de-Shalit 2019. That text builds on earlier work, including Bell DA, de-Shalit A. The Spirit of Cities: Why the Identity of a City Matters in a Global Age. Princeton: Princeton University Press; 2011 .

36. These examples combine facts in news stories and data above with philosophical principles from sources above.

37.
Ibid. The news articles in notes 13 and 14 and philosophical sources in notes 22 and 35 are especially notable.

38. CBC News. COVID-19 in Quebec: Armed forces members arrive to help in long-term care homes. *CBC News* 2020 Apr 18; available at https://www.cbc.ca/news/canada/montreal/covid-19-april-18-1.5537249 (last accessed 14 May 2020).

39. Compare note 18.

40. Again, see the sources in note 22—and the additions in note 35.

41. See, for example, note 14, Duhigg 2020.

42. Note for example, how these principles are all applied in pre-pandemic works cited above in notes 22, 28, 30, 35, etc.

43. This is why federal “emergency” powers are so common despite the cases for municipal control in the sources in notes 22 and 35. Even those who promote “subsidiarity” as a principle for allocating primary authority over many topics to states—or, see note 22, Weinstock’s case, cities—hold that the principle only allocates powers at the most local level that is capable of addressing the subject. Federal emergency powers are thus thought to be justified if and where lower levels of government cannot address them. For more on subsidiarity, see for example, the authors in note 3, Fleming, Levy 2014 or Barber NW. *The Principles of Constitutionalism.* Oxford: Oxford University Press; 2018 at c 7.

44. Recall notes 29 and 30 and surrounding.

45. The sorting, discussed in Rodden J. *Why Cities Lose: The Deep Roots of the Urban-Rural Political Divide.* New York, NY: Basic Books; 2019, could support municipal control. Yet it could also raise concerns about “elite” capture of core policy areas and further politicize emergency-related policy, especially in the present context where “sanctuary cities” remain an unfortunate source of political polarization. Recent protests against distancing measures by more conservative persons at least give an air of reality to this concern. Providing greater power to cities could even undermine compliance with rules within cities, further underling the case for municipal control. After all, providing more power to cities here arguably provides conservative city-dwellers with less input into relevant policies, or at least fewer representatives in the decision-making process who share their basic values and possess enough decisionmaking authority to ensure some decisions reflect those values. This arguably undermines the democratic case for municipal control and reallocation in these circumstances may also lead to noncompliance.

46.

Davies B , Savulescu J. Solidarity and responsibility in health care. Public Health Ethics 2019;12(2):133–443138430210.1093/phe/phz008PMC6655468.

47. See note 46, Davies, Savulescu 2019 at 135, building on Alena Buyx’s earlier work with Barbara Prainsack and with Peter GN West-Oram. For more on solidarity, including work on its implications for social policy, see Banting K , Kymlicka W , eds. The Strains of Commitment: The Political Sources of Solidarity in Diverse Societies. Oxford: Oxford University Press; 2017 .

48. Susan Cutter provides classic statements on why “emergency” is not a natural kind and how even natural disasters are only emergencies following certain government actions in, for example, Cutter SL . Vulnerability to environmental hazards. Progress in Human Geography 1996;20(4):529–539 and a widely-cited collaboration, Cutter SL , Boruff BJ , Shirley WL . Social vulnerability to environmental hazards. Social Science Quarterly 2003;84(2):242–261 .

49. For overviews of these issues, see, for example, the sources in note 3.

50. Recall for example, note 31. It is worth noting that even many of the analyses of healthcare federalism are part of larger works on social policy and federalism, like Greer SL , Elliott H , eds. Federalism and Social Policy. Ann Arbor: University of Michigan Press; 2019 . But healthcare federalism remains an important area of inquiry with its own classics.

51. See, for example, most authors in Anand S , Sen A , eds. Public Health, Ethics, and Equity. Oxford: Oxford University Press; 2005 . Work on the allocation of authority between global and domestic actors is, however, more common. See work by scholars in the same volume or any good global health justice or international health ethics collection for examples.

52.

Parijs PV . Just health care in a pluri-national country In: Anand S , Sen A , eds. Public Health, Ethics, and Equity. Oxford: Oxford University Press; 2005 at 63–180. MacKay D , Danis M. Federalism and responsibility for health care. Public Affairs Quarterly 2016;30(1):1–29 . Gluck AR , Huberfeld N. What is federalism in health care for? Stanford LR 2019;70(6):1689–1803 30203949 provides a legal analysis that engages with federalism theory, but it is heavily focused on recent American experiences.

53. There is also good empirical work on these issues, though this work too often focuses on health policy as an aspect of social policy; for example, Béland D , Lecours A. Nationalism and Social Policy. Oxford: Oxford University Press; 2008 .

54. On COVID-19, as a possible shock allowing reallocation in intergovernmental relations, if not formal powers, see for example, Béland D, Lecours A, Paquet M, Tombe T. A critical juncture in fiscal federalism? Canada’s response to COVID-19. *Canadian Journal of Political Science* 2020;Online First: 1–5.

55. Nascent work on federalism post-COVID-19 largely continues to focus on empirical and legal questions. As with the earlier authority allocations literature cited above, much of this work is by lawyers and political scientists. Much of it is also very good, but the need for additional philosophical reflection is still clear. For a list of resources that is not health-specific, see https://www.mcgill.ca/federalism/federalism-covid-19-pandemic.

